# Assessment of the Efficacy of ReoGo-J Robotic Training Against Other Rehabilitation Therapies for Upper-Limb Hemiplegia After Stroke: Protocol for a Randomized Controlled Trial

**DOI:** 10.3389/fneur.2018.00730

**Published:** 2018-08-28

**Authors:** Takashi Takebayashi, Kayoko Takahashi, Satoru Amano, Yuki Uchiyama, Masahiko Gosho, Kazuhisa Domen, Kenji Hachisuka

**Affiliations:** ^1^Department of Occupational Therapy, School of Health Science and Social Welfare, Kibi International University, Takahashi, Japan; ^2^Department of Rehabilitation, School of Allied Health Science, Kitasato University, Sagamihara-shi, Japan; ^3^Department of Rehabilitation, The Hospital of Hyogo College of Medicine, Nishinomiya-shi, Japan; ^4^Department of Rehabilitation, Hyogo College of Medicine, Nishinomiya-shi, Japan; ^5^Department of Biostatistics, Faculty of Medicine, University of Tsukuba, Tsukuba, Japan; ^6^Department of Rehabilitation Medicine, Hyogo College of Medicine, Nishinomiya, Japan; ^7^Kyushu Rosai Hospital, Moji Medical Center, Kita-kyushu-shi, Japan

**Keywords:** stroke, robotics, upper-extremity, paresis, constraint-induced movement therapy

## Abstract

**Background:** Stroke patients experience chronic hemiparesis in their upper extremities leaving negative effects on quality of life. Robotic therapy is one method to recover arm function, but its research is still in its infancy. Research questions of this study is to investigate how to maximize the benefit of robotic therapy using ReoGo-J for arm hemiplegia in chronic stroke patients.

**Methods:** Design of this study is a multi-center parallel group trial following the prospective, randomized, open-label, blinded endpoint (PROBE) study model. Participants and setting will be 120 chronic stroke patients (over 6 months post-stroke) will be randomly allocated to three different rehabilitation protocols. In this study, the control group will receive 20 min of standard rehabilitation (conventional occupational therapy) and 40 min of self-training (i.e., sanding, placing and stretching). The robotic therapy group will receive 20 min of standard rehabilitation and 40 min of robotic therapy using ReoGo®-J device. The combined therapy group will receive 40 min of robotic therapy and 20 min of constraint-induced movement therapy (protocol to improve upper-limb use in ADL suggests). This study employs the Fugl-Meyer Assessment upper-limb score (primary outcome), other arm function measures and the Stroke Impact Scale score will be measured at baseline, 5 and 10 weeks of the treatment phase. In analysis of this study, we use the mixed effects model for repeated measures to compare changes in outcomes between groups at 5 and 10 Weeks. The registration number of this study is UMIN000022509.

**Conclusions:** This study is a feasible, multi-site randomized controlled trial to examine our hypothesis that combined training protocol could maximize the benefit of robotic therapy and best effective therapeutic strategy for patients with upper-limb hemiparesis.

## Introduction

Severe, persistent paresis occurs in over 40% of stroke patients (1) and is reported to significantly decrease their quality of life (2). Thus, much research has been conducted to develop interventions, with many specifically targeting upper extremity hemiplegia. Among the many examples of neuroscience-based rehabilitation (neuro-rehabilitation) strategies, there is strong evidence supporting robotic therapy, constraint-induced movement therapy (CIMT), and task-oriented training (3, 4).

Robotic therapy is considered an effective intervention for mild to severe hemiplegic arm (5, 6), and is cost-effective for chronic stroke patients in terms of both manpower and medical costs (7, 8). However, its effects may be limited for some patients. Some researchers have found that robotic therapy effectively improves arm function as measured by the Fugl-Meyer Assessment (FMA) (9) and Action research arm test (ARAT) (10), but does not improve the use of the affected arm in activities of daily living (ADL) as measured by the Motor activity log (MAL)-14 (11) and by analysis of data from an accelerometer attached to the affected arm (6, 12–14).

On the contrary, CIMT is the most well-established intervention for improving the use of the affected arm in ADL (15). CIMT consists of three components: (1) a repeated task-oriented approach, (2) a behavioral approach to transfer the function gained during training to actual life (also called the “transfer package”), and (3) constraining use of the affected arm. Some researchers consider the transfer package the most important component of CIMT. In fact, research has shown that usage of the affected arm in daily life is significantly different between patients treated with and without the transfer package component (16, 17). However, many therapists question whether CIMT could benefit their patients because of the shortage of sites possessing the clinical resources to provide the intervention for the long duration required for effectiveness (18).

Therefore, there is an urgent need to establish an effective therapeutic approach, especially for upper-limb hemiplegia during the chronic stage of stroke recovery for which there are few clinical resources (In Japan, the insurance system only allows 260 min per month). Therefore, we will compare the efficacy of several therapy methods. As a control, we will monitor changes in arm function in patients undergoing a short, standard rehabilitation by a therapist and standard self-training (control group). This will be compared to similar self-training including robotic therapy with the ReoGo-J device as an adjuvant therapy (RT group). Finally, the robotic therapy will be compared to combined therapy including robotic therapy and CIMT (CT group). Through these comparisons, we will investigate the effect of robotic therapy, both alone and in combination with CIMT, which we hypothesize will complement each other in chronic stroke rehabilitation. Here, we report the structure and protocol of a multi-center, randomized controlled trial.

## Materials and methods

### Objective

This study is intended to verify the increased efficacy of self-training with robotic therapy compared with the commonly practiced traditional occupational therapy on upper-limb motor function and function in ADL in moderate to severe chronic post-stroke upper-limb hemiplegic patients. In addition, another objective of the study is to verify the degree of superiority of combination training including both robotic therapy and CIMT over training with robotic therapy alone in these patients. Therefore, in this study, primary objective is to compare the RT and control group, secondary objective is to compare the RT and the CT group. Additionally, to compare the CT and control group is auxiliary analysis. We hypothesize that robotic therapy will improve recovery over the usual care alone, and that combining robotic therapy with CIMT will further enhance its benefits.

### Study design

This study will be conducted as a multi-center, prospective, randomized, parallel group study. We aimed to enroll 120 chronic stroke patients from approval by the institutional review board until March 31, 2017. The chronic stroke patients enrolled will be randomized to three groups: a control group to receive conventional occupational therapy (usual care) and self-training that follows the concept of usual care; a group to receive usual care and self-training with robotic therapy; and a group to receive combination training that includes task-oriented training aimed at improving function in ADL and robotic therapy.

The principal investigator will register the study on the clinical trial registration system of the University Hospital Medical Information Network (UMIN000022509) after approval of this study by the clinical research institutional review board of the Hyogo College of Medicine (#2248) and before enrolling subjects.

### Settings

The settings for this study include multiple hospitals and clinics throughout Japan. All sites provide outpatient rehabilitation for stroke patients at the chronic stage of recovery.

### Recruitment

All patients will be screened for the inclusion and exclusion criteria described below. After the patient is determined to meet the criteria, the physician will obtain informed consent from the patient.

### Inclusion and exclusion criteria

Patients will be considered eligible for this study if they are between 20 and 80 years of age, have chronic upper-limb hemiplegia from a supratentorial stroke that occurred at least 6 months before enrollment, are undergoing outpatient or ambulatory rehabilitation, and meet the following functional score requirements: FMA upper-limb scale (9) score of less than 44 points, Stroke Impairment Assessment Set (19) upper-limb distal function score of 1b or above, and modified Ashworth scale (MAS) (20) score of 2 or less.

Patients will be evaluated upon enrollment by their physician, and those with multiple strokes or a cerebellar/brain stem stroke, who have extreme upper-limb pain, or whose upper-limb function is deemed to be improving are not eligible for the study. To avoid complication with other medical conditions, patients with neuromuscular diseases, malignant tumors, balance or gait disturbance, or other serious uncontrolled diseases will be excluded. To ensure patients have the cognitive ability to participate in training and evaluation, patients will be excluded if they have serious aphasia or higher cortical dysfunction (a lack of lucid decision-making ability to participate this study, or a score of 24 points or less on the mini-mental state examination 21). Possible confounding effects of other treatments will be avoided by excluding patients who have received intensive training with an upper-limb training robot or constraint-induced therapy for upper-limb hemiplegia at any time after their stroke, or a botulinum toxin injection within 16 weeks of enrollment. Additionally, before decision-making a participation of study, patients received the standard rehabilitation (physical and occupational therapy) who are not excluded in this study because (1) in study of rehabilitation area for chronic stroke patients, there are few studies that excluse patients receiving the standard rehabilitation before participation of the study from the participant (22–24); (2) in Japan which have health-insurance system that covers all of its citizens, there are few stroke patients in the chronic stroke phase who have not received physical therapy, if they are excluded, the feasibility of this study falls significantly. However, after decision-making a participation of this study, patients were forbidden to receive any other rehabilitation, out of study.

### Withdrawal from study

Patients will be withdrawn from the study if they undergo certain prohibited therapies after enrolling in the study. These include functional electrical stimulation, transcranial magnetic stimulation, and transcranial direct current stimulation. Training with any other upper-limb rehabilitation robot is also prohibited after informed consent. Patients must not receive botulinum toxin injections during the treatment period.

The investigator can decide to withdraw an enrolled and allocated patient from this study if they have difficulty beginning or continuing study participation, such as failure to attend study visits, withdrawal of informed consent, adverse events or complications, recovery of arm function so that no further rehabilitation is needed, or any other event judged by the investigator to warrant withdrawal. The investigators, after withdrawing a subject, shall perform the planned end-of-study examinations to the extent possible and record the observations, last date of training, and reasons for withdrawal on the case report form.

### Enrollment and randomization

Subjects who meet all of the inclusion criteria and none of the exclusion criteria, and have given informed consent, will be evaluated by the investigators for enrollment. At enrollment, information will be collected including demographic data, body measurements, stroke information, complications, lifestyle factors, and baseline function measures. The investigator will verify that the patient is eligible to participate and enter information into the electronic data capture and web allocation system as required.

Randomization will be done through the web allocation system. The patients will be randomized to one of the three treatment groups using dynamic allocation by the minimization method based on the baseline FMA upper-limb score, center, period after stroke, and age.

### Blinding

This study integrates the prospective, randomized, open-label, blinded endpoint (PROBE) study model (25). Because the training methods differ between groups, it is not feasible to blind the subjects, therapists present at the training, and physicians to the treatment. Therefore, the individuals involved in endpoint evaluation, rather than those involved in the treatment, will be blinded. The primary endpoint (FMA) and one of the secondary endpoints (ARAT) will be assessed remotely through audio and visual evaluations of video footage that has undergone blinding. The remaining assessments involve each subject's subjective evaluation, and therefore cannot be blinded, and simple palpation, which will be performed by physicians or therapists who were not present at the training to ensure objectivity.

### Interventions

All patients will receive training 3 times weekly for 10 weeks. During each training session, patients in the control arm of the study will receive 40 min of self-training (sanding, placing, stretching, and repetitive reaching/grasping/releasing practice) and 20 min of usual care (conventional occupational therapy including stretching, joint range-of-motion exercises, correct-movement exercises, and ADL exercises). The RT group intervention will consist of the same 20 min of usual care, and 40 min of self-training with the ReoGo®-J upper-limb rehabilitation device (certification No. 226AHBZX00029000). The CT group intervention will include 40 min of self-training with robotic therapy and 20 min of CIMT training (shaping, task-practice, and transfer package).

### Outcomes

All outcomes will be measured at baseline, 5 and 10 weeks. The primary outcome examined will be changes in the FMA upper-limb score (9). This assessment examines motor and joint function, balance, and sensation in hemiplegic patients and results are represented as a numerical score with a maximum of 66 points.

Secondary outcomes include changes in: (1) the “amount of use” and “quality of movement” components of the MAL-14 scale (11), which assesses limb function in ADL; (2) the individual components of the FMA (9); (3) the ARAT score, another measure of upper-limb function (10); (4) the motricity index (26), an assessment of muscle strength in stroke patients; (5) the MAS score (20), which assesses muscle tone (i.e., spasticity); (6) the active range of motion of the shoulder, elbow, forearm, wrist, and fingers (27); and (7) the Stroke Impact Scale (28), a quality of life assessment scale for stroke patients.

Safety outcomes will include the occurrence of any adverse events. Adverse events will be assessed as either related or unrelated to the study treatment, and their severity graded using the CTCAE Ver 4.03 (29) as a reference. All adverse events will be recorded on the medical chart and case report form. In the case of serious adverse events, including those that are life-threatening or result in hospitalization, a “serious adverse event and malfunction report” will be prepared by the investigator and submitted to the hospital director promptly.

### Data monitoring and management

The research director is responsible for monitoring the study, including ensuring that the data reported by the investigators are accurate, complete, and verifiable in comparison with the source documents and other records. An operating procedure for monitoring will be prepared by the principal investigator in advance, and the principal investigator shall appoint monitors to conduct this monitoring as appropriate. At each hospital, the investigator and the hospital director shall cooperate with all investigations by a monitor, the institutional review board, or a regulatory authority. The investigator and director must present all study-related records, such as source documents, when requested by the investigating body.

Because this study uses a medical device that has received marketing approval, together with the nature of the interventions and the sample size, specific quality assurance audits do not need to be planned. Rather, routine data monitoring as described above will ensure data quality.

The electronic case report form will be provided by the data center of the Tsukuba Clinical Research and Development Organization (T-CReDO). The data center will also perform quality control of the collected data, specifically by conducting logical checks. In cases where inconsistencies, missing values, or other issues are detected in the entered data, the investigator will be queried, and they will revise the electronic case report form if necessary. After completion of data quality control, a case review will be convened. The data center will create a dataset for analysis and transfer quality control records and datasets to the study statistician.

The investigator and director of each hospital are responsible for retaining all records, and the data center will retain the anonymized, transcribed case report form data. These data will be retained for 5 years after completion or termination of the study or 3 years after the last publication of results from the study, whichever is later, unless an individual study site has established a longer retention period.

### Confidentiality

Data such as subject information and data collected from observations and examinations will be stored as an anonymized, linkable dataset using identification codes within the data center. Each participating study site shall manage the coding keys linking identification codes to respective subjects within its facility. All individuals involved in the study will take care to protect subjects' identifying information when handling documents, anonymizing case report forms, and incorporating data into publications.

### Data access and dissemination

Subjects in this study may obtain or access the protocol and study-related documents by making a request to the treating physician, provided that doing so would not hinder the protection of personal information of other subjects or the assurance of originality of this study. After study completion, the principal investigator shall organize and publish the results promptly in an academic journal or academic conference, among other media. The ownership of any papers or presentations prepared using the data collected in this study shall be decided through consultations with the principal investigator. Any copyright will be shared by the lead author and coauthors.

### Sample size

The principal objective of the study is to verify the superiority of the RT group over the control group in the improvement of FMA upper-limb score. Using the minimal clinically important difference (MCID) as a reference, the mean difference between the patients in the RT and control groups with respect to the FMA upper-limb score is expected to be 4.25 points (30). Given that the subjects in this study will be chronic patients, the deviation is expected to be smaller than that in the pilot study conducted in recovering patients (standard deviation, 8.8) (6), and thus a standard deviation of 6 is expected. Based on a two-sided significance level of 0.05 and a power of 0.9 on Student's *t*-test, a sample size of 39 per group is needed. Considering some loss to follow-up, the sample size was increased from 39 to 40 per group. Additionally, in our similar previous study (6), we recruited 56 case in 6 facilities (about nine case per a facility). Therefore, considering difficulty to recruited case in chronic stage, we judged this study will be feasible to request more than 20 facilities to participate this study.

### Statistical analyses

Two analysis populations will be established: the safety analysis set and the efficacy analysis set. The safety analysis set will include all patients who were allocated, and the numbers and percentages of safety endpoints will be compared between groups by Fisher's exact test.

The subject characteristics collected upon enrollment will be analyzed by intergroup comparisons of continuous variables by analysis of variance and of categorical variables by Fisher's exact test.

The efficacy analysis set will include all patients for whom primary endpoint data are available based on intention-to-treat principle. Using the mixed effects model for repeated measures, we will perform intergroup comparisons of changes in the FMA upper-limb score at 5 and 10 Weeks of the treatment phase. This model will include the group, time point, group-by-time interaction term, baseline FMA upper-limb score, sex, and botulinum toxin injection. For multiplicity adjustment, Dunnett's test will be employed with the RT group used as a reference. The same analysis will be conducted for the secondary outcomes. The level of significance is set at 0.05.

## Discussion

Here, we describe an intervention protocol to improve upper-limb function in chronic stroke patients experiencing hemiplegia, which we are examining through a multi-center randomized clinical trial. This study is unique and innovative in the areas of neuro-rehabilitation, physical therapy, occupational therapy, and rehabilitation psychology. When establishing new interventions—whether they are drugs, medical techniques, neuro-rehabilitation techniques, or others—investigators must follow established scientific processes for building evidence. These processes begin with a discovery phase, followed by a pre-clinical trial, and finally several phases (I to IV) of clinical trials. The ReoGo-J study described here is a mid-sized phase IV randomized controlled clinical trial with two purposes: (1) to examine the efficacy of robotic therapy, and (2) to determine whether combined therapy consisting of CIMT and robotic therapy is an ideal rehabilitation protocol for chronic stroke patients. Furthermore, our study results could provide insight into whether the arm function gained by robotic therapy is generalizable to actual life.

Examining the efficacy of robotic therapy, there have been only a few studies conducted around the world to examine the efficacy of robotic therapy for patients in the chronic stage of stroke recovery (7, 16, 31–34). Hardly any of this research was conducted in East Asia, and thus our study would also be novel in this respect. The robot used in this study, ReoGo-J, is the successor of ReoGo, which was shown to be effective during stroke recovery (6). This study would be the first randomized controlled trial using this new robotic system.

Our combined therapy protocol could be an effective option for therapists in clinical settings. Our program is especially unique in that patients can gain the positive effects of both CIMT and robotic therapy, and favorable interaction effect: CIMT could complement the weakness of robotic therapy, which is the limited transfer of arm function gained to daily life, and robotic therapy could complement the weakness of CIMT, which needs large clinical resources. Therefore, by demonstrating the efficacy of our new program including training using ReoGo-J, we could establish a feasible robotic therapy protocol that could be easily carried out with limited clinical resources to treat chronic phase stroke patients.

For the each of the various measures we will use to assess rehabilitation, the MCID has been established. Of the measures that quantify general hemiplegic arm function, the MCID for FMA is 4.25–7.25 points (30), and that of the ARAT is 10% of the total score (10), or 5.7 points (35). The MCID of MAL, which measures the use of the affected arm in actual life, is 0.5 points for the amount of use scale (36) and 1.0–1.1 points for the quality of movement scale (37). Because clinical resources are limited, there are few interventions/approaches to achieve MCID in the measures of arm function and arm use. However, our pilot studies have shown that combined robot and CIMT therapy could improve arm function and use of the affected arm, and those increases were greater than MCID (38, 39). Those results suggest we will see a promising effect of CT in this ReoGo-J study, and that the combined therapy will overcome the weakness of robotic therapy and significantly increase the use of affected arm in real-life settings.

Currently in Japan, many elderly stroke patients receive one-on-one rehabilitation by physical or occupational therapist 20 min per day at an adult day-care center run by the long-term care insurance system. The protocol of the control group in this study is expected to represent the conventional rehabilitation usually done at such adult day-care center. Therefore, if this study proves RT or CT group exceeded control group, we could suggest a better protocol for rehabilitation for upper extremity hemiparesis for chronic stroke patients at adult day-care center. Furthermore, our suggestion may increase the recovery of upper extremity function and use of affected arm at the same resources and cost as conventional protocol.

However, there are some limitations in this study. For example, there are many factors affecting the prognosis of upper extremity hemiplegia caused by stroke: age, sex, baseline upper extremity motor function, time after stroke, degree of sensory deficit, patients' degree of motivation, family support, various approaches within robotic therapy and CIMT (i.e., types of movement, repetitions, clinical site). In this study, the allocation factor will be set based on previous studies to control for differences in the following characteristics between groups: (1) baseline FMA score (40), (2) time after stroke (41–43), (3) age (43, 44), and (4) clinical site. However, the other factors cannot be controlled by this method.

Another limitation is that we cannot blind the patient to their allocated intervention. To minimize the bias of placebo effect, we are modeling our study after the PROBE study design. However, in rehabilitation research, it is often impossible to blind patients to the intervention because we cannot provide sham intervention. Therefore, such studies cannot exclude bias caused by the placebo effect.

## Author contributions

TT and KT contributed equally in the study design, writing and reviewing the manuscript. MG contributed in the analysis design. SA, YU, KD, and KH contributed to reviewing the manuscript.

### Conflict of interest statement

TT and KT receive medical consultant fee from Teijin Pharma Limited. The remaining authors declare that the research was conducted in the absence of any commercial or financial relationships that could be construed as a potential conflict of interest.
